# Exploiting the Dynamics of Soft Materials for Machine Learning

**DOI:** 10.1089/soro.2017.0075

**Published:** 2018-06-01

**Authors:** Kohei Nakajima, Helmut Hauser, Tao Li, Rolf Pfeifer

**Affiliations:** ^1^ JST, PRESTO, Saitama, Japan.; ^2^ Department of Mechano-Informatics, Graduate School of Information Science and Technology, The University of Tokyo, Tokyo, Japan.; ^3^ Department of Engineering Mathematics, University of Bristol, Bristol, United Kingdom.; ^4^ Department of Engineering and Information Technology, Bern University of Applied Sciences, Biel, Switzerland.; ^5^ Department of Informatics, University of Zurich, Zurich, Switzerland.

**Keywords:** physical reservoir computing, soft robotics, physical computation, octopus

## Abstract

Soft materials are increasingly utilized for various purposes in many engineering applications. These materials have been shown to perform a number of functions that were previously difficult to implement using rigid materials. Here, we argue that the diverse dynamics generated by actuating soft materials can be effectively used for machine learning purposes. This is demonstrated using a soft silicone arm through a technique of multiplexing, which enables the rich transient dynamics of the soft materials to be fully exploited as a computational resource. The computational performance of the soft silicone arm is examined through two standard benchmark tasks. Results show that the soft arm compares well to or even outperforms conventional machine learning techniques under multiple conditions. We then demonstrate that this system can be used for the sensory time series prediction problem for the soft arm itself, which suggests its immediate applicability to a real-world machine learning problem. Our approach, on the one hand, represents a radical departure from traditional computational methods, whereas on the other hand, it fits nicely into a more general perspective of computation by way of exploiting the properties of physical materials in the real world.

## Introduction

Soft materials have been attracting attention because they add unprecedented functionality to machines and devices. This functionality enables soft materials to be used in a vast array of applications, such as grasping objects,^1,2^ human–robot interactions,^3^ medical and surgical tools,^4^ and prosthetics and wearables.^5^ The inherent softness of such materials results in increased adaptivity and decreased damage to other surfaces during contact.^6,7^ In addition, robots made with soft materials are able to generate complex behaviors with simpler actuations by partially outsourcing control to the morphological and material properties,^8^ which enhances the active coupling between control, body, and environment.^9,10^

Compared with rigid materials, soft materials exhibit rich dynamics including a variety of properties, such as nonlinearity, elasticity, and high dimensionality. In this article, we demonstrate that these dynamic properties constitute an asset that can be effectively employed for machine learning purposes. Our approach is based on a technique called reservoir computing,^11–13^ which is a framework rooted in recurrent neural network learning. When a high-dimensional dynamical system, which is referred to as the reservoir, is driven with input streams, it generates transient dynamics that operate as a type of temporal and finite kernel that facilitates the separation of the input states. If the dynamics involve short-term memory and nonlinear processing of the input stream, then nonlinear dynamical systems can be learned by adjusting a linear, static readout from the high-dimensional state space of the reservoir.

We exploit the rich physical dynamics of soft materials directly as a reservoir for temporal machine learning problems. This is clearly demonstrated using the physical dynamics of a soft silicone arm. Previously, using the same platform, we illustrated the potential of the approach by emulating Boolean functions and nonlinear dynamical systems and by embedding a closed-loop control into the arm without any external memory and nonlinearity support from the controller.^14,15^ The current study builds on these approaches and aims to boost the computational performance of the arm for real-world applications. We use the technique to supply a huge number of computational nodes, making use of the timescale difference between input–output computation and the physical dynamics of the arm. We first evaluate the effectiveness of our approach through two benchmark tasks, and then we show the validity of the approach and its limitations by comparing it with standard machine learning systems. Next, we show that our approach can be readily used for a sensory time series prediction of the arm, which suggests that it can be applied to the detection of anomalies or the interpolation of real-world sensory time series.

## Materials and Methods: Platform and Information Processing Scheme

### Soft body dynamics

**FIG. 1.**
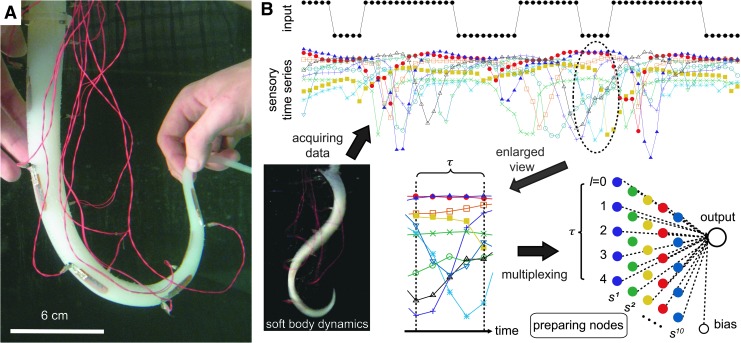
Information processing scheme that uses the dynamics of the soft silicone arm and a multiplexing technique. **(A)** A soft silicone arm immersed in water with 10 bend sensors embedded. The body dynamics of the arm are generated by rotating the arm's base, and the corresponding sensory time series is exploited as a computational resource. *Red wires*, which connect the sensors to a sensory board, were attached as carefully as possible so as not to affect the arm's behavior. **(B)** Schematics summarizing how to prepare virtual nodes according to parameter $\tau$. Using the generated $( 10 \times \tau ) + 1$ node (one bias node) with $w_{out}^i$, the system output is generated. The figure shows the case for $\tau = 5$.

The platform consists of a soft silicone arm, its actuation and sensing systems, and data processing through a PC (see Supplementary Data for details; Supplementary Data are available online at www.liebertpub.com/soro). The soft silicone arm was developed in Ref.^14^ and exhibits diverse body dynamics^14,15^ (Fig. 1A). It is cone shaped, immersed in fresh water, and designed not to touch the ground or walls of the water tank during movement. A similar type of morphology and material characteristics is commonly seen in soft robotic arm design (see, e.g., Refs.^16–18^). For example, for octopus swimming robots, the passive body dynamics of which generate sufficient driving force when actuated appropriately.^19^

The base of the arm can rotate left and right through the actuation of a servo motor (see Supplementary Data for details). The motor commands sent from the PC control the position of the base rotation of the arm. It can take one of two real values, $\{  - 1.0 , 1.0 \}$, where $- 1.0$ corresponds to one maximum rotation angle of the servo motor and $1.0$ corresponds to the other maximum rotation angle of the servo motor. The two maximum rotation angles are symmetrical along a vertical centerline, which is defined by the rotation when the arm is aligned vertically to the water's surface. These two angles are experimentally determined to prevent damage to the motor components. Our system is a typical underactuated system that has only one active degree of freedom but a higher number of passive degrees of freedom in the silicone arm.

The arm contains 10 bend sensors embedded near the surface of the silicone material with their ventral sides directed outward. Each bend sensor has a base value when it is straight, and the ventral side of sensors has a layer of bend-sensitive ink. The sensory value becomes lower than the base value if the sensor bends to the ventral side, and the value becomes higher if it bends to the dorsal side (see Supplementary Fig. S1). In either case, the change in value reflects the degree of the bend. The sensors are numbered from the base toward the tip as *s*^1^ through ${s^{10}}$ and are embedded alternately. The odd-numbered sensors (*s*^1^, *s*^3^, *s*^5^, *s*^7^, and *s*^9^) are embedded on one side of the arm, and the even-numbered sensors (*s*^2^, *s*^4^, *s*^6^, *s*^8^, and ${s^{10}}$) on the other. The unit of the timestep *t* that expresses the time evolution of the system (sensory time series) corresponds to the single sensing and actuation loop of the PC, which is ∼0.03 [*s*] in physical time. Further details on the platform setup are given in Supplementary Data.

### Computational scheme

We demonstrate that sensory time series, which are reflections of the physical dynamics generated by the interaction between the water and the soft silicone material, are readily available as a computational resource for temporal machine learning tasks. In our experiments, the inputs to the system are provided as motor commands, and the corresponding outputs are generated by the weighted sum of the sensory values, which act as computational nodes in our setup.

Previously, using the same platform, we showed that soft body dynamics have a computational performance comparable to the standard reservoir computing network (i.e., the echo state network, ESN), which has the same number of computational nodes as the sensors.^14,15^ In these studies, for each input–output computation, a single corresponding set of sensory values (i.e., 10 values with 1 bias) was used as reservoir states.

Here, we supply an additional number of computational nodes by introducing a different timescale between the dynamics of the soft silicone arm (sensory time series) and the input–output computation (Fig. 1B) to boost the computational power for applications. We set parameter $\tau$ to regulate the timescale of each input–output computation, in which a single computation takes $\tau$ timesteps (see Supplementary Fig. S2). Setting the input–output computational timestep as *k*, the corresponding data series of the *m*-th sensor is $\{  s_{k \tau }^m , s_{k \tau + 1}^m , \ldots , s_{k \tau + l}^m , \ldots , s_{k \tau + ( \tau - 1 ) }^m \}$, where *l* indicates a serial number ranging from 0 to $\tau - 1$ sampled over all the sensors (i.e., from $m = 1$ to 10) for each computational timestep. Using these sampled data, $10 \times \tau$ computational nodes $\{  x_k^1 , x_k^2 , \ldots , x_k^i , \ldots , x_k^{10 \tau } \}$ in total were prepared with reconfigured numbering $i = 10 ( m - 1 ) + l$, which is determined by *m* and *l* (Fig. 1B). This procedure is called a *multiplexing* technique in this article, and this setting of nodes allows us to make full use of the transient dynamics of a physical soft body. Recently, this approach of preparing computational nodes has been applied in different forms of physical reservoirs (see, e.g., Refs.^20–22^). We here, for the first time, apply this scheme for soft materials.

According to the inputs *u_k_* provided to the system as motor commands, the corresponding output *y_k_* is calculated as follows:

\begin{align*}
{y_k} = \mathop \sum \limits_{i = 0}^{10 \tau } w_{out}^ix_k^i , \tag{1}
\end{align*}

where $x_k^0$ is a constant value bias set as “1,” which means we exploit $( 10 \times \tau ) + 1$ in total for computation, and $w_{out}^i$ is the readout weight of the *i*-th computational node (Fig. 1B). In the reservoir computing framework, the learning of the target function ${ \hat y_k} = f ( {u_k} , {u_{k - 1}} , \ldots )$ is conducted by adjusting the linear readout weights $w_{out}^i$. During the training phase of the weights, the input stream is provided to the system, which then generates the arm motions, and the corresponding sensory time series is collected together with the target outputs for supervised learning (see Supplementary Fig. S3). In this article, we apply a ridge regression, which is known as an $L2$ regularization, to obtain the optimal weights (Supplementary Fig. S4). This was introduced to prevent the over-fitting caused by an increase in the computational nodes regulated by $\tau$. The performance of the system output with the optimal weights was then evaluated by comparing it with the target output for a new input stream to demonstrate the generalization property. We also analyzed the effective degrees of freedom of the computational nodes, which provide information about the effectiveness of the physical dynamics to perform given tasks. Further details on the training and evaluation procedures and on the analysis of the effective degrees of freedom are given in Supplementary Data.

## Results: Demonstrations of Temporal Learning Tasks

To demonstrate the computational power of the soft silicone arm, we first used two benchmark tasks proposed in the context of machine learning. The first task is the emulation of nonlinear dynamical systems, called *nonlinear autoregressive–moving-average* (NARMA) systems. The second task is the Boolean function emulation over a binary input sequence. Each task requires a certain amount of nonlinear processing of input and short-term memory of the recent input stream to be implemented. To perform the tasks successfully with our setup, the required memory and nonlinear processing of the inputs will have to be provided through the properties of the soft silicone arm and from the resulting dynamics because we are only adding a static and linear readout.

Throughout this study, the input stream *u_k_* provided as a motor command is set as a random binary sequence of $\{  - 1.0 , 1.0 \}$, which does not add additional temporal coherence originating from the external input to the system and enables us to evaluate the computational power contributed by our system alone. To characterize the contribution of the arm dynamics explicitly, we compare the task performance with that of a simple linear regression (LR) model, ${y_k} = w_{LR}^1 \times {u_k} + w_{LR}^0$, where $w_{LR}^0$ and $w_{LR}^1$ are the readout weights. Note that this LR system corresponds to the case in which no physical body is available, and only the raw input remains for LR. From this comparison, for any system performance better than that of this model, we can conclude that soft body dynamics contribute to task performance positively. The computational power of the arm is further characterized by comparing the task performance of our system with that of a conventional reservoir system, that is, an ESN.^11^ To show the relevance of the multiplexing technique clearly, a setting without multiplexing is also demonstrated, in which we take 10 sensory values, $\{  s_{k \tau + ( \tau - 1 ) }^1 , \ldots , s_{k \tau + ( \tau - 1 ) }^{10} \}$, and 1 bias term as a computational resource for each computational timestep *k* (this setting was applied in our previous experiments^14^). The learning and evaluation of these systems are conducted with the same scheme using the same time series/conditions as that of our soft silicone arm to make a fair comparison (see Supplementary Data for the detailed settings of the ESN).

### NARMA task

The NARMA task is frequently used as a benchmark in the context of recurrent neural network learning to evaluate whether the system can implement nonlinear computations with long time lags. The first NARMA system is the second-order nonlinear dynamical system, which can be written as follows:

\begin{align*}
{y_k} = 0.4{y_{k - 1}} + 0.4{y_{k - 1}}{y_{k - 2}} + 0.6u_k^3 + 0.1. \tag{2}
\end{align*}

This system was introduced in Ref.^23^ and used, for example, in Refs.^15,24,25^ We call this system NARMA2 in this article. The second NARMA system is the *n*th-order nonlinear dynamical system, which can be written as follows:

\begin{align*}
{y_k} = \alpha {y_{k - 1}} + \beta {y_{k -  1}} ( \mathop \sum
\limits_{j = 0}^{n - 1} {y_{k - j - 1}} ) + \gamma {u_{k - n
+1}}{u_k} + \delta , \tag{3}
\end{align*}

where $( \alpha , \beta , \gamma , \delta )$ are set to $( 0.3 , 0.05 , 1.5 , 0.1 )$. Here, *n* is varied using values from 3 to 10, and the corresponding systems are called NARMA3 to NARMA10, respectively. In particular, NARMA10 with this parameter setting was introduced in Ref.^23^ and is commonly used (see, e.g., Refs.^13,15,25^). Here, according to the input stream, the system should simultaneously emulate all the NARMA systems, which we call multitasking.

**FIG. 2.**
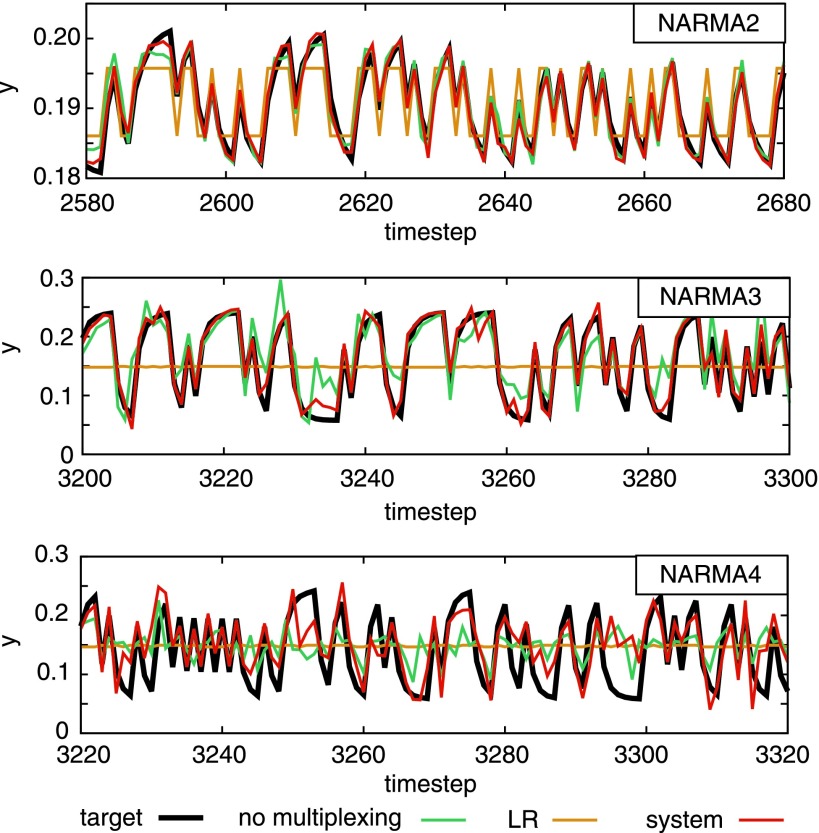
Typical performances for the NARMA tasks in the evaluation phase. In all the shown examples, the performance of the LR system and the system performance without multiplexing (labeled as “no multiplexing”) are overlaid as a reference. In the *upper*, *middle*, and *lower diagram*, the system performance for the NARMA2, NARMA3, and NARMA4 tasks is shown with $\tau = 20 , 10 ,$ and 11, respectively. See texts for details. LR, linear regression; NARMA, nonlinear autoregressive–moving-average.

Figure 2 plots typical examples of the task performance of NARMA2, NARMA3, and NARMA4, each with different settings of parameter $\tau$. Clearly, in each NARMA task, the outputs generated by our system trace the target outputs more accurately than the LR system does. Furthermore, the system performance with a multiplexing technique is clearly superior to that without it, which suggests the effectiveness of the technique. This can be confirmed by checking Supplementary Video S1.

**FIG. 3.**
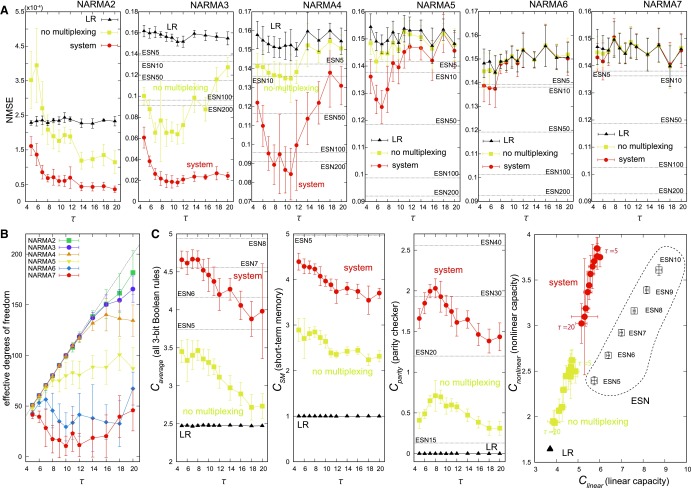
Performance analyses of the NARMA tasks and the Boolean function emulation tasks. **(A)** Plots showing the averaged NMSEs according to $\tau$ of each NARMA task (NARMA2–7). **(B)** The averaged effective degrees of freedom of the system to perform each NARMA task are presented according to $\tau$. The *gray line* presents $f ( \tau ) = 10* \tau + 1$, which represents the maximum degree of freedom. **(C)** Plots showing the averaged capacities of the Boolean function emulation tasks. The first plot from the *left* shows the total capacity ${C_{total}}$, which is the capacity averaged over all the three-bit Boolean function emulation tasks according to $\tau$. The second and third plots from the *left* show the capacities ${C_{SM}}$ and ${C_{parity}}$ according to $\tau$, which are for the short-term memory task and the parity check task representing a typical linear and nonlinear task, respectively. The diagram at the *right* end shows ${C_{nonlinear}}$ compared with ${C_{linear}}$, which overlays the results for $\tau = 5 - 20$. As a reference, some plots contain the averaged results of the LR system, the system performance without multiplexing (“no multiplexing”), and the performance of the ESN. Note that “ESN10,” for example, indicates the results of the ESN with 10 nodes. For all the plots, the error bars show the standard deviations. ESN, echo state network; NMSE, normalized mean squared error.

Detailed analyses for the task performance from NARMA2 to NARMA7 in terms of averaged normalized mean squared error (NMSE) are shown in Figure 3A. We can see that the performance of our system is much better than that of both the LR system and the system without multiplexing, especially when the order of the target NARMA system is small. According to the increase of the order of the target NARMA system, the performance of our systems (including the system without the multiplexing) approaches that of the LR system step by step. Actually, for the NARMA7 task or larger order tasks, we can confirm that the performance of our systems becomes equivalent to that of the LR system for all the $\tau$ settings (see Supplementary Fig. S4 in Supplementary Data for the results of the larger order tasks other than NARMA7).

Interestingly, for each NARMA task, the value of $\tau$ that shows the best performance was different. For example, for the NARMA2 task, the case when the value of $\tau$ is relatively large, such as around $\tau = 20$, showed the best performance. For the NARMA3 and NARMA4 tasks, the case for value around $\tau = 7 - 12$ showed the best performance; for the NARMA5 and NARMA6 tasks, the cases for value around $\tau = 6 - 8$ and value around $\tau = 5 - 7$, respectively, showed the best performance; and for the NARMA7 task, the case for value around $\tau = 5 - 6$ showed the best performance. Note that parameter $\tau$ not only regulates the number of computational nodes but also controls the behavior of the arm, and this indicates that how the input information processed through the body dynamics is different (see Supplementary Fig. S2). As a result, the computational power that we can induce from the arm will also differ according to $\tau$. In particular, for the NARMA3 and NARMA4 tasks, it is clear from the figure that our system can induce higher or comparable computational power than the conventional ESN having 200 computational nodes, which is a larger number of computational nodes than our system with multiplexing. According to the analyses of the effective degrees of freedom of the system to perform each NARMA task, the lower the order of the target NARMA system is, the better the system exploits the increasing number computational nodes according to the increase of $\tau$ (Fig. 3B). This result supports the effectiveness and suggests the range of validity of the multiplexing technique.

### Boolean function emulation tasks and computational capacity

Emulation tasks of Boolean function against random binary input stream are also popular benchmark tasks to evaluate the power of computational systems (see, e.g., Ref.^26^). In this article, we applied the emulation task of a three-bit Boolean function expressed as ${y_k} = f ( {u_{k - 2 - d}} , {u_{k - 1 - d}} , {u_{k - d}} )$, where *f* is a Boolean function and *d* is a delay and can vary from 0 to 49. According to the increase of the number of *d*, more memory for the previous input is needed to emulate the function. There are ${2^3} = 8$ input patterns and ${2^{{2^3}}} = 256$ functions, and these functions include both linear and nonlinear functions.^27^

To evaluate the task performance for each target function, a measure *capacity C* is used (e.g., Ref.^28^). By calculating the square of correlation between the system output and the target output for given delay *d*, *C* is expressed as a summation of the calculated squared correlation over all the delays from 0 to 49. Note that, unlike in the case of NMSE, if the system output emulates the target output well over the delays, the value of the capacity becomes higher. We exploit several variants of this measure for the analyses. See Supplementary Data for details on the measures.

First, to see the overall performance of our system, we analyzed the averaged capacity over all the three-bit Boolean functions ${C_{average}}$ (we have excluded functions that outputs only 0 or 1 from the analyses because the results are trivial). Figure 3C (left diagram) plots the results of the averaged ${C_{average}}$ for each $\tau$, showing that ${C_{average}}$ takes the highest value when $\tau$ is around 5–8. In general, our system performance for Boolean function emulation tasks was not as good as the performance of an ESN having the same number of computational nodes. Actually, these capacity values when $\tau$ is around 5–8 are comparable with those of an ESN with 7–8 computational nodes. Nevertheless, our system showed much higher values of ${C_{average}}$ than the LR system and the system without multiplexing over all the values of $\tau$, suggesting the effectiveness of the multiplexing technique.

To further characterize the computational power of our system, we analyzed the capacities for two target Boolean functions as representatives of linear and nonlinear functions. The first is a function that outputs a previous input value as ${y_k} = {u_{k - d - 2}}$, which represents a linear function commonly used to evaluate the system's short-term memory.^26,28^ The second is a famous parity checker function, which represents a nonlinear function. Capacities for these two functions are denoted as ${C_{SM}}$ and ${C_{parity}}$, respectively, for clarity. The results for ${C_{SM}}$ and ${C_{parity}}$ according to $\tau$ are shown in Figure 3C (middle diagrams). Clearly, for both cases, our system outperforms the cases for the LR system and the system without multiplexing. For ${C_{SM}}$, we can see that our system takes the maximum value of ${C_{SM}}$ at $\tau = 5$, and it gradually decreases until the value of $\tau$ gets to 12, and takes the fixed value for the increasing value of $\tau$. For ${C_{parity}}$, it has a peak value at $\tau = 8$. These results suggest that according to the selection of $\tau$, the type of computational power that can be exploited from the arm is different, which we have also confirmed from the analyses of the NARMA task. In particular, for ${C_{parity}}$, the peak value is comparable with the value of an ESN having 30–40 computational nodes.

By calculating the averaged capacity over all the linear functions ${C_{linear}}$ and that over all the nonlinear functions ${C_{nonlinear}}$, we illustrated the relationship between these two capacities in Figure 3C (right diagram). There are two important points that we can observe from the plot. First, we can see that according to the introduction of a time-multiplexing technique, both ${C_{linear}}$ and ${C_{nonlinear}}$ improve significantly, but the relationship (ratio) between them does not change. Second, we can see that our system has a tendency to take the ratio of ${C_{nonlinear}}$ to ${C_{linear}}$ higher than the ESN, which characterizes the property of the type of computation that can be induced from our system.

## Sensory Time Series Predictions

As we have demonstrated so far through two benchmark tasks, the dynamics of the soft silicone arm can be used for temporal learning tasks, which has the potential to implement a comparable computational performance with a conventional ESN. In particular, we have revealed that there exists a preference regarding the type of computation for the system according to parameter $\tau$, which is considered to be regulated by the balancing between the increase of computational nodes according to the multiplexing technique and the corresponding type of dynamics the soft body generates. Here, we aim to perform a machine learning task using the soft silicone arm for engineering applications.

In general, sensory time series carry important information about the state of the body and are essential ingredients in deciding the next motion of robots or motor commands in the sensorimotor loop. In real-world applications, however, these sensory values are often fluctuated against external noise. They may accidentally stop sensing and break down due to external damage, which causes not only the loss of data but also has severe effects on overall behavioral generation. To face these situations, one solution would be to construct a model of the sensory time series of the robot under a certain behavior using machine learning. This would allow us to interpolate the missing data and also implement an anomaly detection scheme in sensory time series (by calculating the deviation from the modeled sensory value). Needless to say, in the case of soft robots, sensory time series are, in general, diverse containing memory of previous motor commands and nonlinear processing of them. Hence, they are not able to predict without using a temporal machine learning scheme, such as recurrent neural networks.

Here, we aim to implement a sensory time series prediction using our scheme. That is, we predict the sensory value of sensor *i* at computational timestep *k*, exploiting the other sensory time series, which means the target output is expressed as ${y_k} = s_{k \tau + \tau - 1}^i$. Assuming that sensor *i* is missing, the output is calculated with the weighted sum over $9 \times \tau + 1$ computational nodes prepared using the rest of the nine sensors using the same procedures of multiplexing explained previously. The optimal weights are also trained using ridge regression. See Supplementary Data for details on the experimental settings. We also compare our system performance with that of the LR system and the ESN, the detailed settings of which are given in Supplementary Data.

**FIG. 4.**
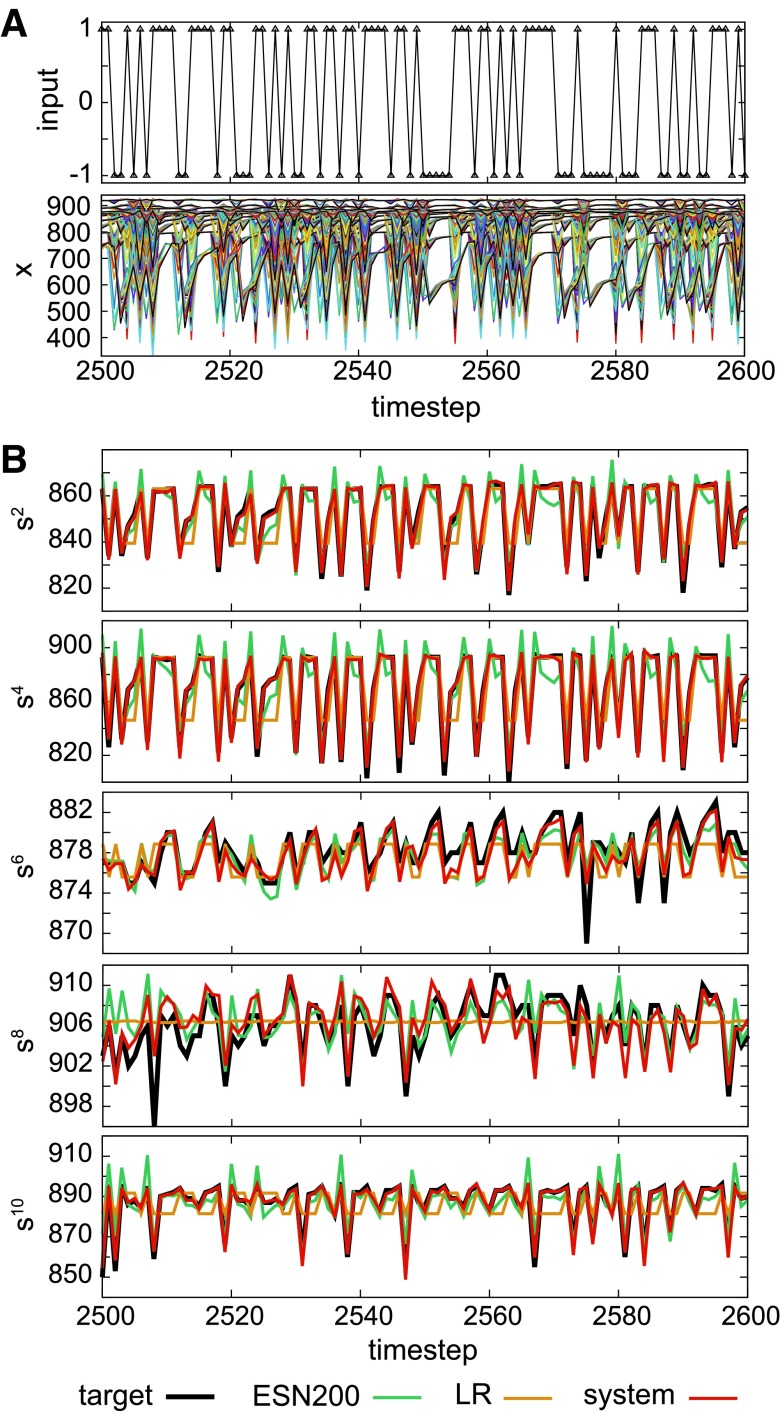
Typical performances of the sensory time series prediction tasks in the evaluation phase. **(A)** Random input sequence *u_k_* (*upper diagram*) and the corresponding sensory time series $x_k^i$ (*lower diagram*) during the task performance. All the sensory time series are overlaid in the *lower plot*. **(B)** In all the shown examples, the performance of the LR system and the performance of the ESN with 200 nodes (ESN200) are overlaid as a reference. From the *upper* to the *lower diagram*, the target sensory time series is from ${s^2} , {s^4} , {s^6} , {s^8}$ to ${s^{10}}$. Parameter $\tau$ is set to 16.

Figure 4 shows typical examples of the performance for the sensory time series prediction task. We can observe that each target sensor shows characteristic temporal patterns, and for each case, our system output traces the target sensory time series more precisely than the LR system and the ESN that has 200 computational nodes (see also Supplementary Fig. S5 and Supplementary Video S2).

**FIG. 5.**
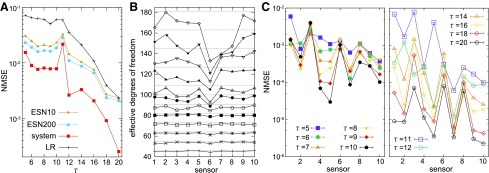
Performance analyses for the sensory time series prediction task. **(A)** The averaged NMSE_*total*_ of each $\tau$ setting is plotted (see Supplementary Table S1 for details). The performances of the LR system, ESN10, and ESN200 in terms of the averaged NMSE_*total*_ are also plotted for comparison. Note that the *y*-axis is in logarithmic scale. **(B)** The averaged effective degrees of freedom analyzed for the prediction tasks according to the target sensor number are shown. The results for all the experimental $\tau$ settings are overlaid. **(C)** The averaged NMSE according to the target sensor number is plotted. The results for $\tau = 5 - 10$ are overlaid in the *left diagram*, and those for $\tau = 11 - 20$ are overlaid in the *right diagram*. In both plots, the *y*-axes are in logarithmic scale with the same range.

Figure 5A shows the results of NMSE_*total*_ according to $\tau$. The measure NMSE_*total*_ is a sum of the NMSE calculated for the case of all the 10 targeted sensors, and it represents the overall performance of the system with the given $\tau$ (see Supplementary Data). First of all, we can clearly confirm that our system outperforms the LR system and the ESNs that have 10 and 200 computational nodes in all the settings of $\tau$. In general, NMSE_*total*_ has a tendency to decrease by increasing the value of $\tau$ (especially when $\tau$ gets larger than 12). Note that because the target sensory time series changes according to $\tau$, even if the system construction is not related to $\tau$, the performance of the LR system and the ESN can change accordingly. This suggests that the target sensory time series are becoming easy to predict in general, according to the increase of parameter $\tau$. For the case of $\tau = 11$, the NMSE_*total*_ of our system shows higher value together with that of the ESNs that have 10 and 200 computational nodes, implying that the target sensory time series themselves are more difficult to predict.

Next, for each case of the target sensor, we analyzed the effective degrees of freedom of the reservoir constructed by the rest of the sensory time series (Fig. 5B). Interestingly, for some target sensors, the effective degrees of freedom increase according to the increase of the value $\tau$, but for some target sensors, such as *s^6^*, the effective degrees of freedom do not increase accordingly. This implies that even if the value of $\tau$ increases and the computational nodes increase accordingly, the useful information for the prediction of the target sensory time series was not included in the rest of the sensory time series. This result not only characterizes the structure of the reservoir when each target sensor was set but also reveals the relationship between the target sensor and the rest of the sensors in terms of predictability. Note, however, that the results for the effective degrees of freedom and the task performance do not always correspond (e.g., it is easy to imagine, depending on the type of task, the same performance is achievable with reservoirs having different degrees of freedom).

Figure 5C plots the averaged NMSE by varying the target sensor number for each setting of $\tau$. We can see that some sensors are relatively easy to predict and others are very difficult. Furthermore, the prediction of some sensors can be more accurate by increasing the value of $\tau$ (e.g., for ${s^4} , {s^5}$, and *s*^7^ when $\tau$ is around 5–10 or for *s*^9^ and ${s^{10}}$ when $\tau$ is around 11–20), but others were not influenced by the increase of $\tau$ (e.g., for *s*^3^ when $\tau$ is around 5–10 or for *s*^6^ and *s*^8^ across all the experimented $\tau$). We can also confirm that the high value of $NMS{E_{total}}$ at $\tau = 11$ is mainly due to the bad prediction performance of the target sensor *s*^5^. See also Supplementary Figure S6 for details.

In summary, our results suggest that to predict the target sensory time series of the soft body, it is more effective to exploit the rest of the sensory time series, which are already present, as a computational resource than to construct a predictive model from scratch using the target input/output relationship. This suggests that the useful information of some sensory time series for prediction is flowing into the other sensors using the physical soft body dynamics through time (similar observations were reported in Ref.^29^). By increasing the value of $\tau$, not only are the sensory time series used as a computational resource but also the target sensory time series are changed; therefore, we can speculate that the balancing of several factors is important to characterize the prediction performance.

## Conclusion

In this article, we have demonstrated that the dynamics of the soft silicone arm itself are readily available when implementing temporal machine learning, which was shown through two benchmark tasks and a sensory time series prediction task. This is realized by introducing different timescales for the input–output and for the physical body dynamics using parameter $\tau$ and by subsequently applying the multiplexing technique, which allows us to effectively boost the computational power by increasing the number of computational nodes.

Our approach outsources the computational load, which is usually executed within the for-loop in the program, to the physical dynamics of the soft material. This can be interpreted as a massive parallel computation executed through the natural soft dynamics, and we can expect energy efficiency as a result. In greater detail, the outsourcing happens in the recruitment of nonlinear kernels that can be linearly combined to estimate a nonlinear dynamical system, and the advantage comes in the ability of the soft arm to encode them with arbitrary memory spans. Furthermore, according to the specific morphology of the physical materials and the specific way of actuation, this scheme can induce significantly high computational power for certain tasks. This suggests that each material has its own computational preference (some material dynamics are good for some tasks but not for other tasks). It would be beneficial to investigate in future how the morphology, material property, actuation patterns, and environmental conditions affect the information processing capability of the system.

Recently, many soft technologies have been developed. For example, in Ref.,^30^ a microfluidic logic regulating on-board fluid flow was introduced to generate autonomous actuation in soft robots. Furthermore, an energetically autonomous soft robot, which acquires energy from an aquatic environment using a soft robotic feeding mechanism, was proposed in Ref.^31^ In terms of soft sensing, many types of flexible sensors have been proposed that do not influence or damage the natural dynamics of the soft body.^32^ In addition, the body morphology can be designed and manufactured using a novel three-dimensional printing technique, designed to work especially with soft materials.^33,34^ By integrating these soft technologies, we expect that our approach presented in this study will further exert its potential to generate novel application domains that strongly interface with information science, including machine learning and artificial intelligence, material science, and physical science.

The dynamical perspective and its exploitation for computation are, in essence, two sides of the same coin. The most standard way of doing computation is by using a modern silicon chip as a computational resource, which can be very conveniently programmed using standard programming languages. The physical resource is the silicon chip itself, which has, like any physical system, particular dynamics. By forcing a “high-gain regime” onto the chip (the voltages), which gives it its digital characteristics, the dynamics are exploited in particular ways that are directly related to computation.

In the area of analog computing, the physical characteristics of the medium are exploited, which makes it potentially fast and cheap but subject to error. Another approach to exploiting the physical properties of chips for computation is field-programmable gate arrays (FPGAs), which are particularly suited for highly specialized applications. The reason they are very fast is that the electronic circuits are *configured* rather than simulated digitally. In other words, the analog electronic circuits represent the physical dynamics used for computation. Mechanical computing systems like ancient calculators are subject to yet a different kind of dynamics (but that is nevertheless exploited for computing). Thus, the idea to search for different kinds of materials, such as soft materials, that might be suitable for certain types of computation and develop ways in which their dynamics can be exploited is, in fact, very natural. However, the idea leads to radically different ways of viewing computation and its relation to materials, and it opens up novel theoretical developments and practical applications.

## Supplementary Material

Supplemental data
