# Supplementary material for: Exploiting the Dynamics of Soft Materials for Machine Learning
Source: Soft Robot. 2018 Jun 1;5(3):339–47. doi: 10.1089/soro.2017.0075 (PMC5995269; doi:10.1089/soro.2017.0075)
Supplement: Supplemental data [file Supp_Data.zip › Supp_Data.pdf]

Supplementary Data

Platform Setups

This section gives detailed information on an experimental platform consisting of a soft silicone arm, its actuation and sensing systems, and a water tank (100 cm long, 50 cm wide, and 50 cm deep) filled with fresh water as the working environment. The soft silicone rubber arm (ECOFLEXTM00-30 from Smooth-On, Inc.) was made using an ABS plastic mold manufactured by a three-dimensional printer. Ten bend sensors were embedded near the surface of the silicone arm during the process of making the arm, during which two separate pieces of the mold were assembled. The sensors were aligned parallel to the arm's surface with an equal distance of 3.2 cm between them. The arm has a cone shape and is 44.7 cm in length, with a radius of 1.4 cm at one end (base) and a radius of 0.15 cm at the other end (tip). Further details on the manufacturing process of the arm can be found in Ref.[1]

During experiments, the arm is actuated by a Dynamixel RX-64 servo motor at the arm's base. The arm's base consists of rigid plastic and is directly connected to the motor. For each experimental trial, the amount of water in the tank is controlled to be the same, which is set at the same height of the apical surface of the plastic material of the base when the arm is aligned vertically to the water's surface. The sensors embedded in the arm are used to detect the amount of bending at each location of the arm during experiments. They are flexible, lightweight bend sensors from Flexpoint Sensor Systems, Inc. The size of each sensor is about 3.2 cm long (including connectors), 0.7 cm wide, and <0.1 cm thick. Each sensor consists of a thin plastic base film, a layer of coated bend-sensitive ink, and two connectors.[2] Further specifications of the sensor and its typical response curves can be found in the design manual of the provider.[3] A sensor board with voltage dividers and a 16-channel multiplexer, an Arduino™ MEGA 2560 board, and a PC were used for our data processing. The motor commands and sensory data, which are sent through the red electrical cable to the board, were recorded at each control timestep for further analysis. Further details on our sensory data acquisition system can be found in Ref.[1]

Supplementary Figure S1 shows the averaged step response curves for normalized sensory time series when switching the motor command from −1 to 1. The plots are obtained from averaging 68 trials of responses. For each trial, the arm is initially relaxed and set at the base rotation −1. The plots clearly represent the range of repeatability of the sensory responses of our system. Moreover, Supplementary Figure S2 shows a plot comparing the behavior of the actual base rotation when the same motor command is sent with different τ settings. Here, parameter τ is set at the minimum and maximum values ($\tau = 5$ and 20, respectively) used in the experiment. It clearly illustrates a behavioral difference induced by parameter τ.

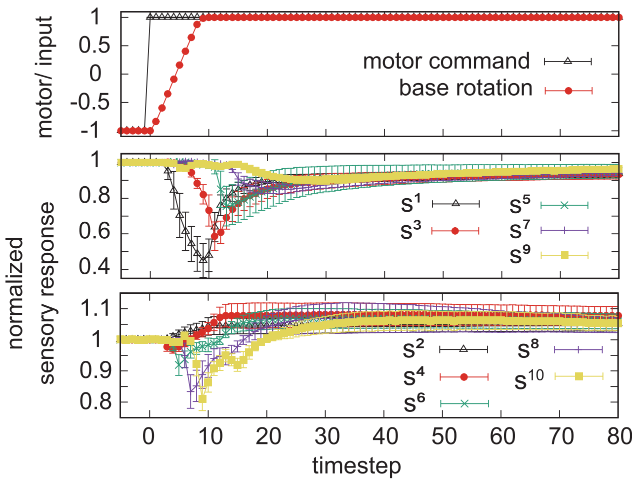

SUPPLEMENTARY FIG. S1. Averaged normalized sensory response curves. Averaged normalized sensory response curves are presented for all the sensors (the odd-numbered sensors are plotted in the *upper diagram*, and the even-numbered sensors are in the *lower diagram*) according to the switching of the motor command from −1 to 1 at timestep 0. The actual base rotation is overlaid with the motor command, exhibiting that it takes nine timesteps to reach from one end of the rotation to the other. The error bars show the standard deviations.

Time Series Preparations for Experimental Procedures

As explained in the main text, we demonstrated that the soft silicone arm can be used for performing two benchmark tasks and one realistic task of sensory time series prediction. For all the tasks, we have varied parameter τ for 5–12, 14, 16, 18, and 20 in the experiments. For each setting of τ, we performed a few dozen trials for the experiment. Namely,

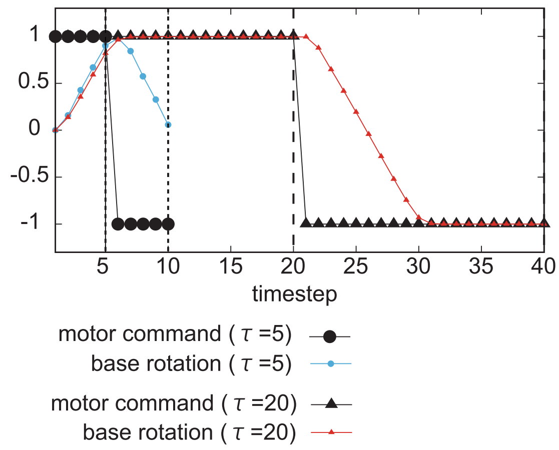

SUPPLEMENTARY FIG. S2. Comparisons of the behavior of the actual base rotation between $\tau = 5$ and $\tau = 20$. For both cases, the same motor commands, "1, − 1," are sent for clear comparisons, and the initial base rotation is set at the *middle* position and relaxed. We can clearly observe that the base rotation of the arm differs according to parameter τ, even if the motor command is the same. Note that the *x*-axis represents the physical timestep of the system.

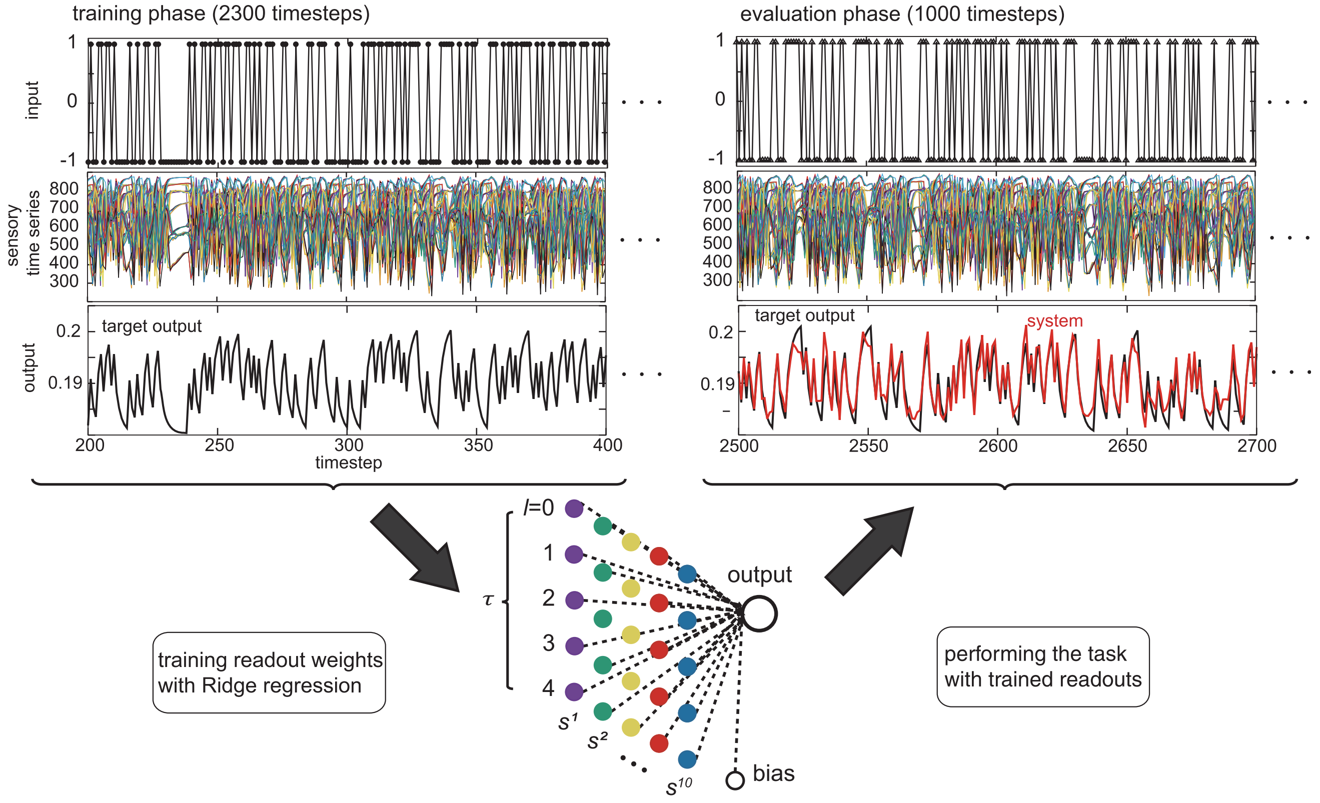

SUPPLEMENTARY FIG. S3. Schematics explaining the training and evaluation procedures in this study. Note that a random input pattern in the evaluation phase is different from that used in the training phase. Plots show an actual example of time series in the training phase and in the evaluation phase (first 200 timesteps are shown, e.g., in both cases). In this example, τ is set to 5. See text for details.

when $\tau = 5, 6, 7, 8, 9, 10, 11, 12, 14, 16, 18,$ and 20, we performed 14, 14, 13, 13, 13, 12, 12, 14, 13, 13, 10, and 12 trials, respectively. The learning scheme of our system is based on supervised learning, and each experimental trial consists of a washout phase (200 timesteps), a training phase (2300 timesteps), and an evaluation phase (1000 timesteps), making 3500 timesteps in total (here, "timestep" is based on the input–output timescale expressed as k). Using the time series data of 2300 timesteps in the training phase, the readout weights are optimized using the ridge regression, which is explained in the next section in detail. By applying a new input time series, the trained readout weights are exploited to calculate the corresponding system output in the evaluation phase, and the performance is evaluated by comparing the system output with the target output (Supplementary Fig. S3).

Ridge Regression and the Effective Degrees of Freedom

In this section, we explain the detailed information on the training procedures of the readout weights used in this study. As explained in the main text, according to the increase of the computational nodes through the multiplexing technique, overfitting should be avoided. Therefore, in this article, we used a ridge regression, which is based on the $L2$ regularization.[4] Ridge regression minimizes the residual sum of squares (RSS) between the system output and the target output in the training phase with a procedure of penalizing the size of the weights. Considering that the training phase is defined in the time region $201 \le k \le 2500$, the optimal weights $\hat{w}^i_{out}$ are defined as the value that minimize $\sum_{k=201}^{2500}(\hat{y}_k - y_k)^2 + \lambda\sum_{i=0}^{10\tau}(w^i_{out})^2$, where λ is a real nonnegative ridge parameter. By collecting the training data set for 2300 timesteps, we have $2300 \times N$ matrix $\mathbf{X}$, where N is a system size. Note that for the nonlinear autoregressive–moving-average (NARMA) task and the Booelan function emulation task, $N = 10\tau + 1$ and for the sensory time series prediction task, $N = 9\tau + 1$. In addition, we collect the corresponding target outputs over time for 2300 timesteps as $\mathbf{y} = [\hat{y}_{201}, \ldots, \hat{y}_{2501}]^T$. Then the optimal output weights, $\hat{\mathbf{W}}_{\mathbf{out}} = [\hat{w}^0_{out}, \ldots, \hat{w}^{N-1}_{out}]^T$, can be obtained by:

$$\hat{\mathbf{W}}_{\mathbf{out}} = (\mathbf{X}^T\mathbf{X} + \lambda\mathbf{I})^{-1}\mathbf{X}^T\mathbf{y}. \quad (9)$$

Next, we should determine the appropriate value of λ. As a basic property related to λ, when the value approaches 0, the optimal weight $\hat{\mathbf{W}}_{\mathbf{out}}$ approaches the weight obtained by the ordinary least squares $\hat{\mathbf{W}}^{\mathbf{OLS}}_{\mathbf{out}} = (\mathbf{X}^T\mathbf{X})^{-1}\mathbf{X}^T\mathbf{y}$, and when the value approaches infinity, the optimal weight $\hat{\mathbf{W}}_{\mathbf{out}}$ approaches 0. For the selection of the value λ, the concept of the degrees of freedom (df) plays an important role. The value df is determined as:

$$df = \sum_i \frac{\lambda_i}{\lambda_i + \lambda}, \quad (10)$$

where $\{\lambda_i\}$ are the eigenvalues of $\mathbf{X}^T\mathbf{X}$, which means that $\{\lambda_i\}$ can be obtained by the training data set. Because df is a

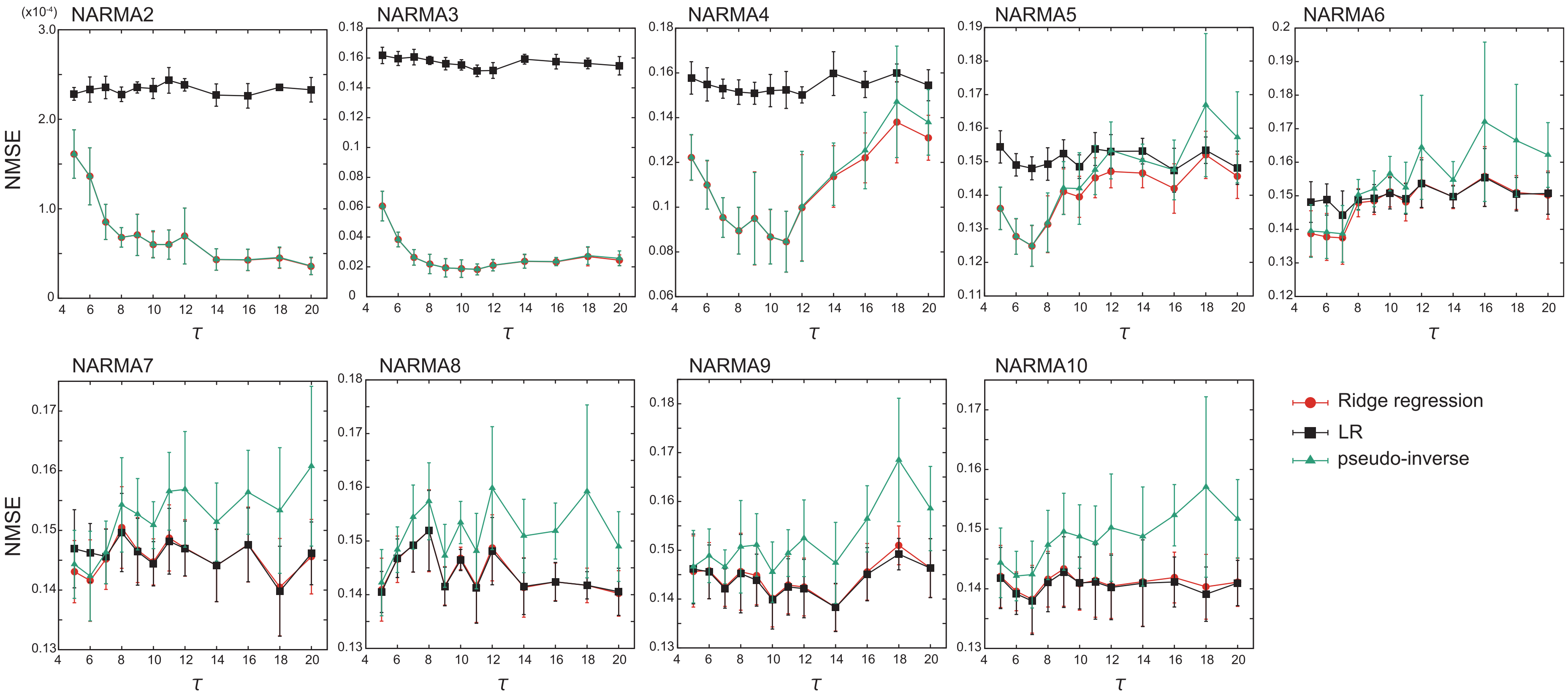

SUPPLEMENTARY FIG. S4. Comparisons between the simple linear regression and the ridge regression in terms of the performance of the NARMA tasks. Plots show the averaged NMSEs according to τ for each NARMA task (NARMA2–10). As references, all plots contain the averaged results for the performance of the LR system, the readout weights of which are trained by the ridge regression. The simple linear regression is implemented by the Moore–Penrose pseudo-inverse technique. NARMA, nonlinear autoregressive–moving-average; LR, linear regression; NMSE, normalized mean squared error.

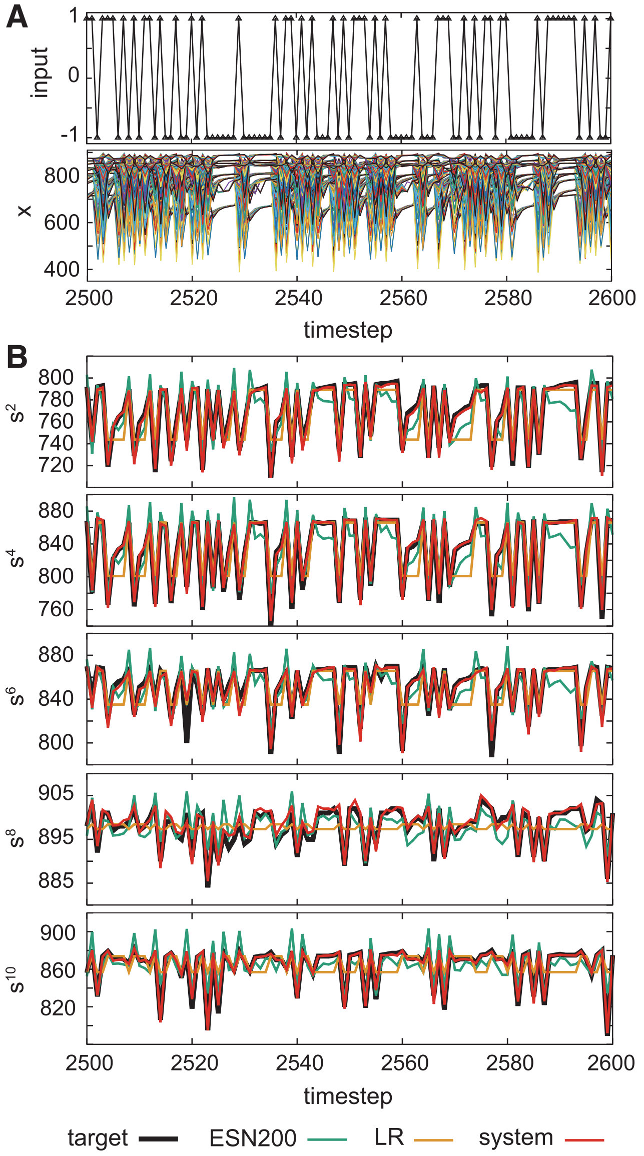

SUPPLEMENTARY FIG. S5. Typical performances of the sensory time series prediction tasks in the evaluation phase. **(A)** Random input sequence u_k (*upper diagram*) and the corresponding sensory time series x_k^i (*lower diagram*) during the task performance. All the sensory time series are overlaid in the lower plot. **(B)** In all the examples shown, the performance of the LR system and the performance of the ESN with 200 nodes (ESN200) are overlaid as a reference. From the *upper* to the *lower diagram*, the target sensory time series is from s^2 to s^4, s^6, s^8, and s^{10}. Parameter τ is set to 16. This trial shows a different trial from Figure 4 in the main text, and although it starts from the same initial configuration of the arm, it is driven by a different random input sequence. ESN, echo state network.

one-to-one correspondence with λ and its maximum value is N, we vary df from 1 to N with increments of 1. Then, for each value of df, obtaining a unique λ_{df} from Equation (10), we can calculate the corresponding $\hat{\mathbf{W}}_{\mathbf{out}}^{\mathbf{df}}$ from Equation (9). By using $\hat{\mathbf{W}}_{\mathbf{out}}^{\mathbf{df}}$ and calculating the residual sum of squares $RSS = \sum_{k=201}^{2500} (\hat{y}_k - y_k)^2$, Akaike's information criterion (AIC) can be obtained as:

$$AIC = M \log(RSS) + 2df, \tag{11}$$

where M is 2300 in our experiment. By calculating AIC for each df, we obtain the optimal λ and $\hat{\mathbf{W}}_{\mathbf{out}}$ that minimizes AIC. The value of df that minimizes AIC in this procedure is called *effective degrees of freedom* in this study.

To check the effectiveness of the ridge regression, using the NARMA task for example, we compared the performance with the ordinary least squares calculated with the Moore–Penrose pseudo-inverse (Supplementary Fig. S4). Supplementary Figure S4 plots the averaged normalized mean squared error (NMSE) for each setting of τ from the NARMA2 task to the NARMA10 task. We can see that when the order of the target NARMA system gets bigger than 5, the system performance using readout weights obtained by the Moore–Penrose pseudo-inverse is becoming worse than that of the linear regression (LR) system. We can speculate that this is caused by overfitting and can confirm that the ridge regression effectively selects the appropriate weights.

Performance Evaluations

For performance evaluations, we calculated the NMSE for the NARMA task and the sensory time series prediction task, expressed as follows:

$$NMSE = \frac{\sum_{k=2501}^{3500} (\hat{y}_k - y_k)^2}{\sum_{k=2501}^{3500} \hat{y}_k^2}, \tag{4}$$

where $\hat{y}_k$ and y_k are the target output and the system output at timestep k, respectively. For each τ setting, NMSEs for all the trials are calculated and averaged for the analysis. In particular, NMSE_{total} defined in the sensory time series prediction task is expressed as follows:

$$NMSE_{total} = \sum_{i=1}^{10} NMSE_i, \tag{5}$$

where NMSE_i is an NMSE obtained when the sensor i was set as a target sensory time series for predictions. Supplementary Table S1 summarizes the averaged NMSE_{total} according to each setting of τ with standard deviations. Supplementary Figure S5 shows the case for $\tau = 16$, in a trial different from that shown in Figure 4, expressing the repeatability of the task performance. The averaged NMSE_i is also analyzed according to the target sensor i for each setting of τ (Supplementary Fig. S6). In general, we can confirm that our system outperforms the LR system and the echo state network (ESN) having 200 computational nodes. We can find several exceptions, for example, when $\tau = 5$ and the target sensor was s^1 and s^2 or when $\tau = 11$ and the target sensor was s^5 (see Supplementary Fig. S6 for details).

For the Boolean function emulation task using the system output time series, in each target function with given delay d, we calculate memory function MF_d expressed as follows:

$$MF_d = \frac{cov^2(y_k, \hat{y}_k)}{\sigma^2(y_k)\sigma^2(\hat{y}_k)}, \tag{6}$$

where $cov(x, y)$ and $\sigma(x)$ express the covariance between x and y and the standard deviation of x, respectively. As

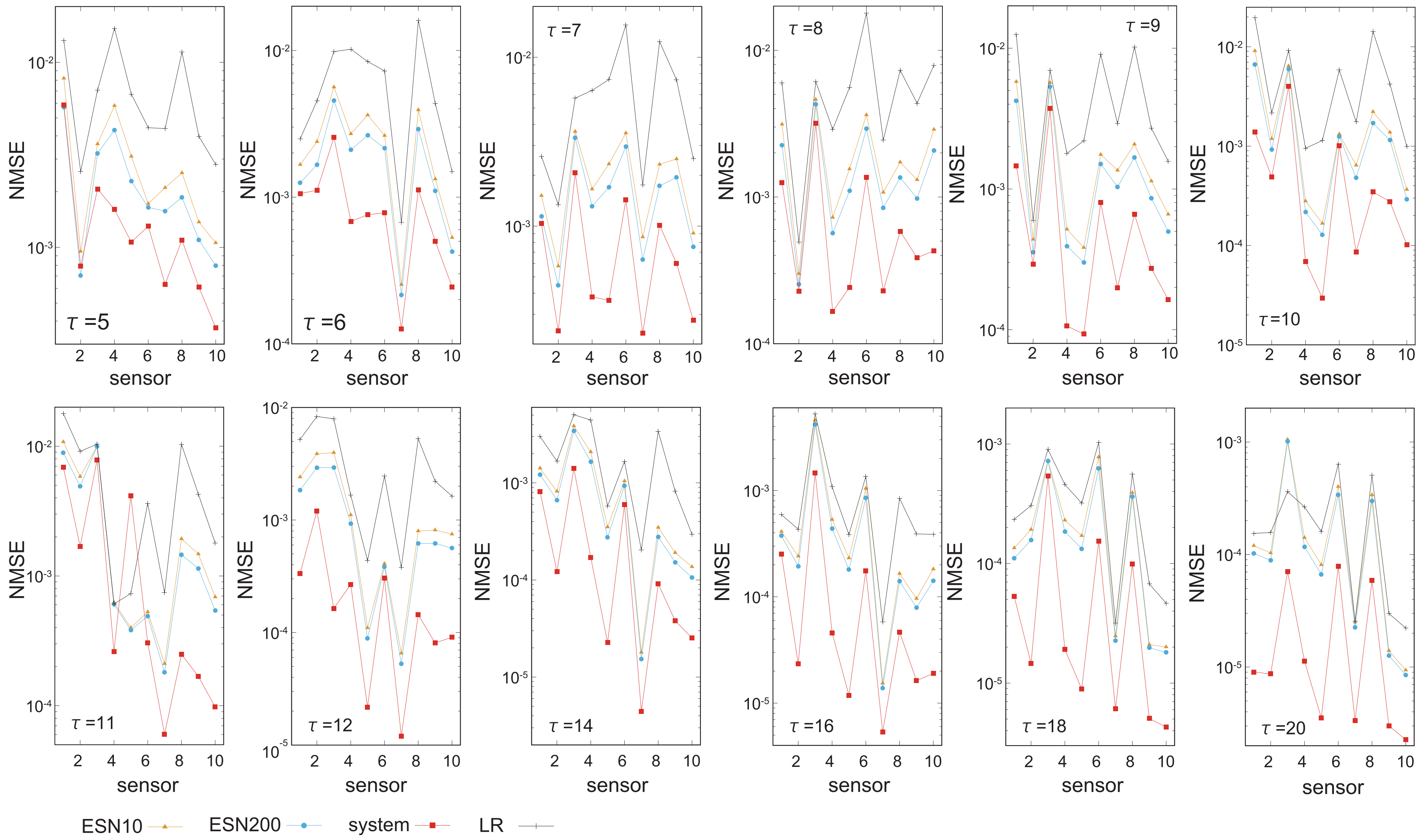

SUPPLEMENTARY FIG. S6. Performance analyses of the sensory time series prediction task for each τ setting. The averaged NMSE according to the target sensor number is plotted for each τ setting. The performances of the LR system, ESN10, and ESN200 in terms of the averaged NMSE are also plotted for comparison. Note that for each plot, the y-axis is in logarithmic scale.

Supplementary Table S1. Analysis of the Sensory Time Series Prediction Tasks in Terms of $NMSE_{TOTAL}$

	System	*LR system*
τ	*Mean* ± *SD*	*Mean* ± *SD*
5	$1.54 \pm 1.29\ (\times 10^{-2})$	$7.17 \pm 6.89\ (\times 10^{-2})$
6	$0.89 \pm 1.14\ (\times 10^{-2})$	$6.52 \pm 6.81\ (\times 10^{-2})$
7	$0.76 \pm 1.00\ (\times 10^{-2})$	$6.29 \pm 6.95\ (\times 10^{-2})$
8	$0.81 \pm 1.16\ (\times 10^{-2})$	$6.10 \pm 6.58\ (\times 10^{-2})$
9	$0.78 \pm 1.19\ (\times 10^{-2})$	$5.05 \pm 5.87\ (\times 10^{-2})$
10	$0.78 \pm 1.22\ (\times 10^{-2})$	$6.01 \pm 6.94\ (\times 10^{-2})$
11	$2.17 \pm 5.30\ (\times 10^{-2})$	$5.95 \pm 5.37\ (\times 10^{-2})$
12	$0.26 \pm 0.20\ (\times 10^{-2})$	$3.55 \pm 3.34\ (\times 10^{-2})$
14	$0.33 \pm 0.66\ (\times 10^{-2})$	$2.11 \pm 3.00\ (\times 10^{-2})$
16	$0.21 \pm 0.55\ (\times 10^{-2})$	$1.08 \pm 2.40\ (\times 10^{-2})$
18	$0.90 \pm 2.10\ (\times 10^{-2})$	$3.95 \pm 6.48\ (\times 10^{-2})$
20	$0.25 \pm 0.54\ (\times 10^{-2})$	$2.32 \pm 3.67\ (\times 10^{-2})$

The averaged $NMSE_{total}$ with the standard deviation is shown for each case.

LR, linear regression; NMSE, normalized mean squared error; SD, standard deviation.

explained in the main text, the three-bit Boolean function has 256 rules, and this rule can be numbered as $rule = \sum_{u_0=0}^{1}\sum_{u_1=0}^{1}\sum_{u_2=0}^{1}(2^{2^0 * u_0 + 2^1 * u_1 + 2^2 * u_2}) * f(u_0, u_1, u_2)$ from $rule = 0$ to $rule = 255$ (note that the input value "-1" is replaced with "0" when calculating the rule number of the function). According to this numbering system, the rule picked as a representative for the linear function, which outputs the previous input value, can be expressed as $rule = 240$. The parity checker function, introduced as a representative for the linear function, is $rule = 150$. We denote the memory function of $rule = r$ with given delay d as MF_d^r. Then the capacity for each rule C_r can be expressed as follows:

$$C_r = \sum_{d=0}^{49} MF_d^r. \tag{7}$$

Here, the measure $C_{average}$ introduced in the main text is defined as:

$$C_{average} = \frac{1}{254}\sum_{r=1}^{254} C_r. \tag{8}$$

Note that, as explained in the main text, $rule = 0$ and $rule = 255$ are excluded from the analyses. Furthermore, the capacities C_{SM} and C_{parity} introduced in the main text are C_{240} and C_{150}, respectively. In a three-bit Boolean function, rules are classified into linear and nonlinear rules in terms of input separability.[5] Linear rules contain 104 rules in total and nonlinear rules contain 152 rules in total. The measures C_{linear} and $C_{nonlinear}$ introduced in the main text are defined as the averaged capacity over all the linear rules and nonlinear rules, respectively. (Note that $rule = 0$ and $rule = 255$ are also excluded from this analysis.) All the capacity measures already defined are calculated for all the trials in each τ setting, and the averaged capacity (and the standard deviation) calculated using all the trials is used for the analysis in Figure 3C in the main text.

ESN Settings for Comparisons

To further characterize the computational power of our system, we compared its task performance with that of a conventional ESN.[6] The ESN is a recurrent neural network that has N internal computational units, input units, and output units. We express the activation of the ith internal unit at timestep k as x_k^i. The weights w^{ij} for the internal network connect the ith unit to the jth unit, and the input weights w_{in}^i connect the input unit to the ith internal unit. Internal units with one bias are connected to the output unit through readout weights w_{out}^i (i.e., $x_k^0 = 1$ and w_{out}^0 for a bias term). The readout weights w_{out}^i are trained using the same procedure explained for each task, whereas the internal weights w^{ij} and the input weights w_{in}^i are randomly assigned from the range $[-1.0, 1.0]$ and the range $[-0.01, 0.01]$, respectively, and were fixed beforehand. The internal and output state transitions are expressed as follows:

$$x_k^i = f\left(\sum_{j=1}^{N} w^{ij} x_{k-1}^j + w_{in}^i u_k\right), \tag{12}$$

$$y_k = \sum_{i=0}^{N} w_{out}^i x_k^i, \tag{13}$$

where $f(x) = \tanh(x)$. To make a fair comparison of the task performance, the I/O setting of the ESN was set to be basically the same as our system for each task. For example, the lengths of the washout, training, and evaluation phases and the evaluation procedures were kept the same. The detailed experimental conditions are given for each of these comparisons hereunder.

For the NARMA task, we first prepared 20 different ESNs. For each ESN, we tested the emulation tasks of all the NARMA systems (NARMA2–7) using a multitasking scheme by varying the spectral radius of the internal weights from 0.1 to 2.0 in 0.1 increments. Each trial was driven by different random binary input sequences, $u_t = \{-1.0, 1.0\}$. We picked the lowest NMSE, which indicates the best performance, among all the trials for each NARMA system and obtained the averaged NMSE for each NARMA system emulation task over 20 different ESNs. These averaged NMSEs were used for comparison.

For the Boolean function emulation task, we first prepared 50 different ESNs with a spectral radius fixed at 0.8. For each ESN, we tested the emulation tasks of three-bit Boolean functions, which contain 254 rules excluding rules 0 and 255, using a multitasking scheme wherein each trial was driven by different random binary input sequences, $u_t = \{-1.0, 1.0\}$. Analyses of the performance were conducted using the same procedures defined earlier, and the averaged capacities over 50 trials were used for comparison.

In the sensory time series prediction task, an ESN is required to predict the state transitions of the target sensor, taking the corresponding binary input sequence $u_t = \{-1.0, 1.0\}$, which is taken from the actual input sequences of soft robotic experiments. Here, we prepared 50 different ESNs, and for each ESN we tested the prediction tasks of 10 sensory time series using a multitasking scheme by varying the spectral radius of the internal weights from 0.1 to 2.0 in 0.1 increments for each trial of the soft robotic experiment with each τ setting. We picked the lowest NMSE, which implies the best performance among all the tested spectral radii, and obtained the averaged NMSE for each trial

of the soft robotic experiment with each τ setting over the 50 different ESNs. These averaged NMSEs were used for comparison.

References

1. Nakajima K, Li T, Hauser H, Pfeifer R. Exploiting short-term memory in soft body dynamics as a computational resource. J R Soc Interface 2014;11:20140437.
2. Flexpoint Sensor Systems, Inc. Mechanical design guide. Available at www.flexpoint.com/technicalDataSheets/mechanicalDesignGuide.pdf (accessed August 31, 2013).
3. Flexpoint Sensor Systems, Inc. Electronic design guide. Available at www.flexpoint.com/technicalDataSheets/electronicDesignGuide.pdf (accessed August 31, 2013).
4. Hastie T, Tibshirani R, Friedman J. The Elements of Statistical Learning: Data Mining, Inference, and Prediction (Springer Series in Statistics, 2nd ed.). New York: Springer-Verlag New York, 2009.
5. Chua LO, Yoon S, Dogaru R. A nonlinear dynamics perspective of Wolfram's new kind of science. Part I: Threshold of complexity. Int J Bifurcat Chaos 2002;12:2655–2766.
6. Jaeger H, Haas H. Harnessing nonlinearity: predicting chaotic systems and saving energy in wireless communication. Science 2004;304:78–80.

SUPPLEMENTARY VIDEO S1. Typical performances for the NARMA tasks and the corresponding arm behaviors in the evaluation phase. The system performances when $\tau = 5$, 11, and 20 for the NARMA2, NARMA3, NARMA4, NARMA5 tasks are shown for example. The performance of the LR system and the system performance without multiplexing (labeled as "no multiplexing") are also shown for comparisons. NARMA, nonlinear autoregressive–moving-average.

SUPPLEMENTARY VIDEO S2. Typical performances of the sensory time series prediction tasks and the corresponding arm behaviors in the evaluation phase. The system performances when $\tau = 5$, 11, and 20 are shown with the target sensory time series defined as s2, s4, s6, s8, and s10, for example. The performance of the LR system is also shown as a reference. LR, linear regression.
